# Spinal Cord Stimulation: Viable Therapeutic Option for Postlaminectomy Syndrome in Elderly Patients

**DOI:** 10.7759/cureus.15675

**Published:** 2021-06-15

**Authors:** Alejandro Hallo, Hector Martínez, Karen E Jácome-Calderón, Mayra Rodríguez

**Affiliations:** 1 Internal Medicine, Hospital de Especialidad Eugenio Espejo, Quito, ECU; 2 Medicine, Universidad Central del Ecuador, Quito, ECU; 3 Anaesthesiology, Hospital de Especialidad Eugenio Espejo, Quito, ECU; 4 Medical coordination, Fundación Cambiando Vidas, Quito, ECU; 5 Neurology, Hospital de Especialidades Eugenio Espejo, Quito, ECU

**Keywords:** chronic pain, spinal cord stimulation

## Abstract

We present a 76-year-old man with chronic back pain refractory to treatment secondary to spinal trauma from a motor vehicle accident 34 years ago. After trauma, multiple interventions were performed due to spinal instability. The patient was diagnosed with the postlaminectomy syndrome. Multimodal analgesia management failed to control our patient’s pain, severely affecting our patient and his family’s quality of life. For these reasons, a spinal cord stimulator was implanted despite our patient age. After four months, our patient presented with significant improvement in his life quality.

## Introduction

Low back pain is a public health problem that increases spinal surgeries [1]. Postlaminectomy syndrome (PLS) occurs in about 60% of patients who undergo spinal surgery and is described as persistent pain that causes functional impairment. The complexity of this syndrome requires multimodal management to optimize results [2]. The first-line treatment for failed back surgery (FBS) is physical therapy and oral pain control medication or minimally invasive procedures such as steroid injections [3]. FBS with refractory pain can be managed with antiepileptics, including gabapentin, pregabalin, antidepressants, and opioids [4]. According to several guidelines, if a satisfactory result has not been obtained after optimal doses, it is recommended to consider surgical options like spinal cord stimulation (SCS). This technique has been used since 1967 and has shown great potential in the definitive management of FBS [3]. According to several studies, 67% of patients who underwent SCS reported pain relief at six months of follow-up [5]. Although better results have been observed in young patients [6], the severe impairment of our patients and their family’s quality of life were the reason to consider the implantation of the SCS.

## Case presentation

**Figure 1:**
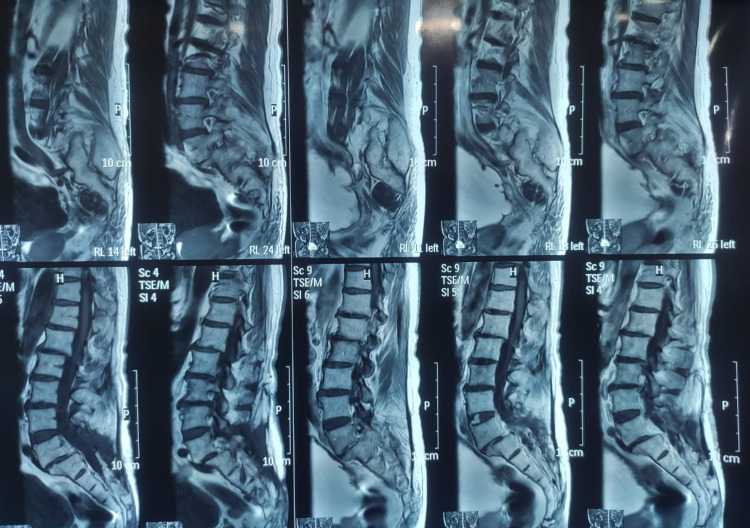
Grade II spondylolisthesis between L5-S1, degenerative spondylopathy in L1-L2 and T12-L1.

We present a 76-year-old man who suffered spinal trauma after a motor vehicle accident 34 years ago. After trauma, medical evaluation immediately showed grade 2 spondylolisthesis with signs of instability at the L5-S1 level. The patient was managed surgically with L5-S1 arthrodesis with intervertebral cage and iliac crest graft. Unfortunately, postoperative infection complicated the patient’s clinical course. For this reason, surgical cleanings were performed for almost six months until infection resolution (Figure 1).

Fifteen years after the trauma, a Harrington bar placement was attempted without success due to extensive fibrosis and bleeding. Initially, the chronic back pain was managed with facet joint injections every three months for three years, without success. He was prescribed 20mg of oxycodone every 8 hours and a ¼ patch (2.5mcg) of buprenorphine with confusion and lethargy reported during the first days of treatment. The initial therapy failed to manage the pain, severely limiting his daily activities. Gabapentin 1200mg and buprenorphine ½ patch (5mcg) every 72 hours. The patient was treated by a psychiatrist for major depressive disorder with amitriptyline and lactulose for moderate constipation. Despite pharmacological pain management, the patient did not show improvement, so he was referred to our hospital for refractory pain management. It was decided to place a spinal cord stimulator.

**Figure 2:**
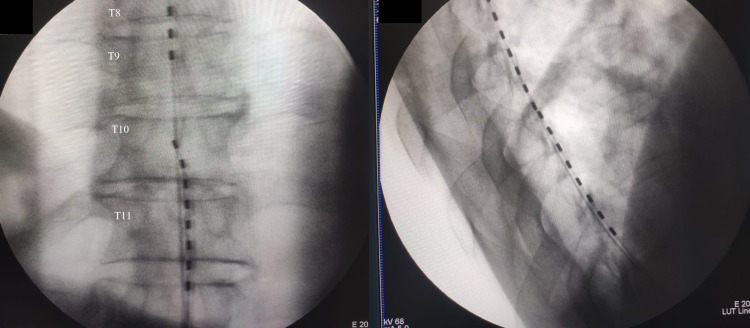
Intraoperative X-ray during spinal electrode placement.

A spinal cord stimulator trial with percutaneous electrodes was placed through L1 and L2 until reaching the thoracic level. During the procedure, abundant fibrosis difficulted access to the thoracic spine; however, it was possible to place electrodes at the thoracic level (Figure 2). Electrode 1: current 5.5 mA, pulse duration 250 µs, frequency 80Hz. Electrode 2: Intensity 6.0mA, pulse duration 350µs, frequency 80Hz.

Due to the considerable clinical improvement of more than 40% with a trial stimulator, a permanent electrode was implanted with similar improvement. Three months later, the patient reported how much the pain decreased. In addition, he reported improvement during the daytime with mild pain, returning to his hobbies, and significant improvement in sleep quality.

## Discussion

Postlaminectomy syndrome occurs when the results of one or more lumbar spine surgeries performed to alleviate pain do not meet the expectations of the patient [2,7]. One of the possible causes of pain is the increased pressure on the pre-and post-vertebral muscles that control the spine’s movement caused by biomechanical changes increasing the load on adjacent structures and causing degenerative changes [3].

Our patient underwent eight spinal surgeries with no successful outcomes, as described in several studies. This result has been observed in up to 40% of patients [3]. According to guidelines recommendations, our patient had been managed for several years with NSAIDs, facet injections, and opioids without relief in pain intensity [3,4,7]. Since chronic opioid use has been associated with high morbidity and mortality and is not associated with better long-term outcomes, it was decided to combine pregabalin, amitriptyline, and buprenorphine [3,7].

Despite being managed at maximal doses, the patient did not obtain adequate pain management. This is an outcome observed in other cases in which it was reported that pharmacological treatment was only helpful in a minority of patients [4]. According to Gatzinsky et al., if a 30% improvement in pain is not achieved or worsens, surgical options should be considered [8].

Spinal cord stimulation has proven to be the most effective treatment in patients with predominantly neuropathic pain in the lower extremities, showing a successful outcome in 62% of patients with FBS [5,7]. The gate control theory of pain by Melzack and Wall proposes that transmission from body to brain is regulated for a mechanism in the dorsal horns of the spinal cord, which acts as a gate depending on the diameters of the peripheral fibres [9]. However, the mechanism for chronic severe pain has not been fully understood. It is hypothesized that the device stimulates the dorsal columns, lateral funiculars, and fibres of the spinal cord’s dorsal roots. This antidromic and orthodromic activation modulates pain perception through the supraspinal and spinal circuits, inhibiting transmission in the ascending nociceptive fibres and increasing the activity of the descending antinociceptive fibres, resulting in pain relief [10].

An electrode is implanted in the posterior epidural space in contact with the spinal cord, with a stimulation frequency range between 20 and 120 Hz [11,12]. It is important to note that pain is not eliminated with SCS but is masked with paresthesia [13]. The implications of living with chronic pain have been associated with a high incidence of work absenteeism, depression, anxiety, deteriorated intra-family relationships, and difficulty performing daily living activities [13]. Our patient developed a severe functional limitation, loss of autonomy, and impairment of his personal and familial relationships. In addition, the significant impact of pain in a patient makes them more vulnerable to depression and anxiety [14]. Older than 65-years-old patients usually are not satisfied with the results of a spinal cord stimulation [6]. However, our patient showed a 50% improvement in pain and a dramatic improvement in his interpersonal and intrafamilial relationships. Medication was also reduced to a ¼ patch (2.5mcg) of buprenorphine and paracetamol on-demand at three months of follow-up. Again, this was an expected outcome, as seen in other studies [5].

## Conclusions

Spinal cord stimulation effectively treats limbs' radicular pain better than more invasive techniques such as reoperation or conservative management. Its use is strongly recommended in multiple conditions such as PLS. Although better results have been observed in patients younger than 65 years-old, SCS can be used within the overall framework of multimodal pain management in patients with severe deterioration in their quality of life.
